# Psychometric evaluation of the Chinese version of the inventory of self-care decision-making styles of older adults: a translation and validation study

**DOI:** 10.3389/fpubh.2026.1944465

**Published:** 2026-10-08

**Authors:** Gaoxiang Zhong, Yan Qiao, Li Zhang, Yanjun Rao, Ting Jiang, Jiao Pu, Zhihui Tang, Su Zhang, Wen Shen, Jiankun Yuan, Haoran Qi, Yangjie Wang

**Affiliations:** 1 Department of Rehabilitation Medicine, Mianyang Central Hospital, Mianyang, Sichuan, China; 2 The Fifth People's Hospital of Chengdu, Chengdu, Sichuan, China; 3 Department of Nursing, Jinzhou Medical University, Jinzhou, China

**Keywords:** chronic disease management, cross-cultural adaptation, healthy aging, older adults, psychometric evaluation, self-care decision-making style

## Abstract

**Background:**

Optimizing self-care decision-making is important for healthy aging, chronic disease management, and health promotion in rapidly aging populations. However, the absence of culturally validated instruments limits the systematic assessment of decision-making styles and the development of person-centered health management strategies for older adults in China. This study aimed to translate and culturally adapt the Inventory of Self-Care Decision-Making Styles of Older Adults (DECIDE) and evaluate its psychometric properties among Chinese older adults.

**Methods:**

This study was conducted in two phases. In phase 1, the DECIDE was translated and cross-culturally adapted into Chinese using Brislin’s forward-backward translation procedure. Expert review and pilot testing were conducted to refine linguistic clarity and cultural relevance. In phase 2, a multicenter cross-sectional validation study was conducted from November 2025 to January 2026. A total of 3,470 older adults were recruited through multicenter convenience sampling from 15 cities in Sichuan, Liaoning, Zhejiang, and Hunan provinces, China. Psychometric evaluation included item analysis, validity assessment, and reliability testing.

**Results:**

All 20 items met the prespecified retention criteria, and the Chinese version retained the four-dimensional structure of the original DECIDE. Content validity was satisfactory, with I-CVI values ranging from 0.800 to 1.000 and an S-CVI of 0.950. Exploratory factor analysis yielded four factors explaining 65.73% of the common variance, and confirmatory factor analysis supported the resulting model (CMIN/DF = 2.513, RMSEA = 0.028). The AVE and CR values ranged from 0.502 to 0.588 and from 0.834 to 0.877, respectively, and the findings supported both convergent and discriminant validity. Cronbach’s α, split-half reliability, and 2-week retest reliability for the total inventory were 0.896, 0.790, and 0.861, respectively.

**Conclusion:**

The C-DECIDE demonstrated satisfactory psychometric properties in this multicenter convenience sample of older adults from four Chinese provinces. It may support the assessment of self-care decision-making styles and inform individualized health education, decision-support strategies, and public health-oriented chronic disease management for older adults.

## 1 Introduction

Population aging and the increasing burden of chronic conditions have placed greater emphasis on older adults’ participation in the daily management of their health (1–3). Older adults routinely make choices concerning symptom monitoring, treatment use, preventive behaviors, rehabilitation, and when to seek professional care (4, 5). Self-care decision-making styles refer to the relatively consistent ways in which individuals approach such health-related choices, including the extent to which they act independently, assume personal responsibility, rely on professional guidance, or neglect their health needs (6–8). These styles describe how individuals approach self-care decisions rather than whether a particular decision is objectively correct. Understanding these patterns may therefore have implications for health communication, decision support, and chronic disease management among older adults (8, 9).

Self-care decision-making styles may be shaped by multiple factors, including functional status, health literacy, social support, family involvement, and characteristics of the healthcare environment (10–14). Assessing these styles is particularly important in older populations, as many older adults must manage multimorbidity, complex treatment regimens, functional limitations, and varying levels of family or professional support (1–3, 11, 12). In community and primary healthcare settings, identifying habitual decision-making patterns may help healthcare professionals tailor communication, health education, and decision support to older adults’ preferences and needs. This information may also support more person-centered approaches to shared decision-making and long-term self-care (9, 15–18).

Despite the potential relevance of this construct, the systematic assessment of self-care decision-making styles among older adults in China remains limited (19). Several available self-care instruments assess disease-specific behaviors, self-management ability, or individual domains such as self-neglect, rather than the broader patterns through which older adults approach health-related decisions (20–24). Recent Chinese psychometric studies have validated instruments for related constructs, including health literacy, social frailty, and health-related quality of life, but these measures do not specifically assess self-care decision-making styles (10, 25, 26). Furthermore, decision-making is embedded within cultural and healthcare contexts. Cultural expectations regarding autonomy, family participation, personal responsibility, and relationships with healthcare professionals may shape how older adults interpret and respond to health-related choices (9, 18, 27). Instruments developed in other populations should therefore undergo appropriate translation, cross-cultural adaptation, and psychometric evaluation before being used among older adults in China.

The Inventory of Self-Care Decision-Making Styles of Older Adults (DECIDE), developed by Matarese et al. (6), was specifically designed to assess self-care decision-making styles among older adults. It comprises 20 items across four dimensions: Independent Decisions, Responsible Decisions, Self-Neglected Decisions, and Guided Decisions. The original validation study reported acceptable psychometric properties. Accordingly, the present study aimed to translate and cross-culturally adapt the DECIDE into Chinese and to evaluate the psychometric properties of the C-DECIDE in a large multicenter sample of older adults in China.

## 2 Materials and methods

### 2.1 Ethics approval and consent to participate

This study was conducted in accordance with the Declaration of Helsinki (28) and was approved by the Ethics Committee of Mianyang Central Hospital (Approval No. S20240253-02). All participants were informed of the study aims, procedures, and confidentiality protections, and written informed consent was obtained prior to participation. Anonymity and privacy were maintained throughout data collection and analysis.

### 2.2 Study design and participants

This methodological study comprised two phases. Phase 1 involved the translation and cross-cultural adaptation of the DECIDE, and Phase 2 comprised a multicenter cross-sectional psychometric validation study conducted from November 2025 to January 2026 at participating hospitals, community health centers, and residential communities in 15 cities across Sichuan, Liaoning, Zhejiang, and Hunan provinces, China.

Participants were recruited using multicenter convenience sampling. At each participating site, trained investigators invited older adults who met the eligibility criteria and were available during the data-collection period to participate. Inclusion criteria were: (1) age ≥ 60 years; (2) ability to communicate effectively with clear consciousness; and (3) willingness to participate with written informed consent. Exclusion criteria were: (1) a documented diagnosis of dementia, severe cognitive impairment, or a psychiatric disorder, or an inability to understand the study instructions and provide coherent responses during initial screening, as judged by trained investigators; (2) hearing or visual impairment severe enough to prevent questionnaire completion; and (3) an acute or terminal illness that precluded participation. Sample size was determined based on recommendations for factor analysis. For EFA, a sample size of about five to ten participants per item is generally advised (29), whereas CFA generally requires at least 200 participants (30). Because EFA and CFA were planned using independent samples, and allowing for an approximately 10% invalid response rate, the minimum required sample size was estimated to be 330. To increase the geographic diversity of the sample and ensure an adequate sample size for psychometric evaluation, recruitment was expanded across multiple sites. In total, 3,600 questionnaires were distributed; 3,516 were returned, and 3,470 were valid, yielding an effective response rate of 96.39%.

### 2.3 Study instruments

#### 2.3.1 The general demographic characteristics questionnaire

The questionnaire was designed based on an expert consultation and literature review. It collected information on sex, age, educational attainment, monthly household income, marital status, residence, living arrangement, and history of chronic diseases.

#### 2.3.2 The inventory of self-care decision-making styles of older adults

Self-care decision-making styles were assessed using the DECIDE, developed by Matarese et al. (6). The DECIDE consists of 20 items organized into four dimensions: Independent Decisions, Responsible Decisions, Self-Neglected Decisions, and Guided Decisions, with five items in each dimension. Each item is rated on a 5-point Likert scale ranging from 1 (“strongly disagree”) to 5 (“strongly agree”). In the original study, McDonald’s omega coefficients for the four dimensions ranged from 0.691 to 0.796 (6).

### 2.4 Translation procedure

Permission to translate and use the original instrument was obtained from the developer, Professor Maria Matarese. The translation followed the Brislin forward-backward translation procedure (31–33). Two bilingual translators independently conducted forward translations (a doctoral candidate in geriatric nursing and a professor of English). Discrepancies were reconciled through discussion to produce a consensus Chinese draft. Two additional bilingual translators then performed back-translation (a geriatric nursing expert and a native English-speaking teacher from the United States). The back-translated version was reviewed by Professor Matarese against the original inventory to verify semantic and conceptual equivalence, resulting in the preliminary Chinese version of the DECIDE.

### 2.5 Cross-cultural adaptation

Cross-cultural adaptation was undertaken using a Delphi expert consultation. Five experts (two geriatric clinical nursing specialists, two geriatric clinical medicine specialists, and one geriatric social medicine specialist) reviewed the translated draft and provided iterative revisions. Experts were eligible if they: (1) had ≥10 years of experience in geriatric care; (2) held an intermediate professional title or above; (3) had a Master’s degree or higher; and (4) provided informed consent to participate. The panel included two men and three women (mean age 47.40 ± 8.29 years) with a mean of 17.60 ± 5.32 years of professional experience. The basic characteristics of the experts are summarized in Table 1.

**Table 1:** Expert basic information.

Expert number	Educational background	Professional title	Research direction	Years of working
P1	Doctoral degree	Senior	Geriatric clinical medicine	16
P2	Doctoral degree	Senior	Geriatric nursing	21
P3	Master’s degree	Intermediate	Geriatric social medicine	12
P4	Doctoral degree	Senior	Geriatric clinical medicine	25
P5	Master’s degree	Intermediate	Geriatric nursing	14

### 2.6 Pilot testing

After expert revision, pilot testing was conducted with 30 older adults to evaluate item comprehensibility, interpretability, and clarity. After completing the questionnaire, participants were invited to provide verbal feedback on item wording, comprehensibility, and consistency with everyday language. The research team reviewed the feedback and made minor wording refinements before finalizing the C-DECIDE for formal psychometric evaluation.

### 2.7 Data collection

Data were collected by trained investigators at participating hospitals, community health centers, and residential communities in 15 cities across Sichuan, Liaoning, Zhejiang, and Hunan provinces. Prior to administration, participants received a standardized explanation of the study purpose and procedures. After written informed consent was obtained, participants completed the anonymous questionnaire independently whenever possible. For participants who experienced difficulty reading the questionnaire but were able to understand the items and provide coherent responses, trained investigators provided assistance by reading the items aloud verbatim and recording the participants’ responses. Completion required approximately 5–10 min, and questionnaires were checked and collected immediately after completion.

### 2.8 Statistical analysis

Data were analyzed using SPSS 26.0 and Amos 24.0. Descriptive statistics were used to summarize participants’ demographic characteristics. Continuous variables were presented as means and standard deviations, and categorical variables were presented as frequencies and percentages. All statistical tests were two-sided, and *p* < 0.05 was considered statistically significant.

#### 2.8.1 Item analysis

For item analysis, the scores of all 20 items were summed to obtain an overall score, which was used to rank the participants. Participants in the upper and lower 27% of the overall score distribution were assigned to the high- and low-score groups, respectively. Independent-samples t tests were conducted to compare the score of each item between the two groups, with the t value used as the critical ratio. Items with non-significant between-group differences or critical ratio values < 3.00 were considered for deletion. Item-total correlations and changes in the Cronbach’s α coefficient of the corresponding subscale after item deletion were also examined. Items were considered for removal if the item-total correlation was non-significant or < 0.40, or if deleting the item increased the Cronbach’s α coefficient of its corresponding subscale (34).

#### 2.8.2 Validity analysis

##### 2.8.2.1 Content validity analysis

Content validity was evaluated by five qualified specialists who rated the relevance of each item. The item-level content validity index (I-CVI) was calculated as the proportion of experts rating an item as 3 or 4. The scale-level content validity index (S-CVI) was computed as the mean of the I-CVI values across all items. Content validity was considered acceptable when I-CVI ≥ 0.78 and S-CVI ≥ 0.90 (35).

##### 2.8.2.2 Construct validity analysis

The full sample was randomly split into two independent subsamples: subsample 1 (*n* = 1,470) for exploratory factor analysis (EFA) and subsample 2 (*n* = 2,000) for confirmatory factor analysis (CFA). For EFA, sampling adequacy was assessed using the Kaiser-Meyer-Olkin (KMO) measure and Bartlett’s test of sphericity. Common factors were extracted using principal axis factoring with Promax oblique rotation, because correlations among the dimensions were theoretically expected (36). Factors with eigenvalues greater than 1 were retained according to the Kaiser criterion. The interpretability of the factor solution and its consistency with the four-dimensional structure of the original instrument were also considered. Items were retained when they had a primary pattern coefficient > 0.40, all secondary pattern coefficients < 0.30, and conceptual consistency with the corresponding factor. Each retained factor was required to contain at least three items (36). Given the five ordered response categories, the large CFA sample, and the absence of substantial item-level nonnormality, the items were treated as approximately continuous, and CFA was conducted using maximum likelihood estimation (37). Model fit was evaluated using chi-square/df (CMIN/DF < 3), root mean square residual (RMR < 0.05), and root mean square error of approximation (RMSEA < 0.08), as well as incremental/absolute fit indices including Tucker-Lewis index (TLI), comparative fit index (CFI), incremental fit index (IFI), goodness-of-fit index (GFI), and adjusted goodness-of-fit index (AGFI), with values > 0.90 indicating acceptable fit (38).

##### 2.8.2.3 Convergent and discriminant validity analysis

The convergent and discriminant validity were evaluated by using average variance extracted (AVE), composite reliability (CR), and the square root of AVE. Thresholds were set at AVE > 0.50, CR > 0.70, and the square root of AVE greater than inter-factor correlations (39).

#### 2.8.3 Reliability analysis

Internal consistency was evaluated using Cronbach’s α and split-half reliability in the total sample. Test–retest reliability was assessed using Pearson correlation coefficients in a subsample of 200 participants who completed the C-DECIDE twice, 2 weeks apart. Reliability coefficients ≥ 0.70 were considered acceptable (40, 41).

## 3 Results

### 3.1 Participant characteristics

A total of 3,470 older adults were included in the study, of whom 1,504 (43.3%) were men and 1,966 (56.7%) were women. The mean age was 75.83 ± 9.54 years, with participants aged 71–80 years constituting the largest age group (41.5%), followed by those aged 60–70 years (37.9%). Most participants had an elementary school education or below (72.3%), reported a monthly household income of 2,000–5,000 yuan (44.4%), were married (75.5%), resided in rural areas (75.8%), and lived with family members (80.4%). In addition, 60.3% of the participants reported having one or more chronic diseases. Detailed characteristics are presented in Table 2.

**Table 2:** Demographic characteristics of the total sample and the EFA and CFA subsamples.

Variables	Group	Total sample (*n* = 3,470) n(%)	EFA subsample (*n* = 1,470) n(%)	CFA subsample (*n* = 2000) n(%)
Sex	Male	1,504 (43.3)	679 (46.2)	825 (41.3)
	Female	1966 (56.7)	791 (53.8)	1,175(58.7)
Age	60–70	1,315 (37.9)	437 (29.7)	878(43.9)
	71–80	1,441 (41.5)	736 (50.1)	705 (35.3)
	81–90	471 (13.6)	166 (11.3)	305(15.3)
	> 90	243 (7.0)	131 (8.9)	112(5.5)
Education level	Elementary school or below	2,508 (72.3)	1,005(68.4)	1,503(75.2)
	Junior high school	489 (14.1)	341(23.2)	148 (7.4)
	Senior high school	366 (10.5)	88 (6.0)	278(13.9)
	Junior college or above	107(3.1)	36 (2.4)	71 (3.5)
Monthly household income	< 2000 yuan	891 (25.7)	556 (37.8)	335(16.8)
	2000–5,000 yuan	1,542 (44.4)	595 (40.5)	947(47.4)
	>5,000 yuan	1,037 (29.9)	319 (21.7)	718(35.8)
Marital status	Married	2,619 (75.5)	1,164 (79.2)	1,455(72.8)
	Never married	401 (11.6)	98 (6.7)	303(15.2)
	Widowed	359 (10.3)	191 (13.0)	168(8.4)
	Divorced	91 (2.6)	17 (1.1)	74 (3.6)
Residence	Rural	2,630 (75.8)	1,057(71.9)	1,573 (78.7)
	Urban	840 (24.2)	413 (28.1)	427(21.3)
Living arrangement	Alone	681 (19.6)	468(31.8)	213(10.7)
	With family members	2,789 (80.4)	1,002(68.2)	1787(89.3)
Chronic diseases	Yes	2092 (60.3)	1,108 (75.4)	984(49.2)
	No	1,378 (39.7)	362(24.6)	1,016(50.8)

### 3.2 Cross-cultural adaptation

Five experts reviewed and refined the Chinese DECIDE draft to enhance clarity and contextual appropriateness. Expert 1 and Expert 4 recommended modifying: “health decision” to “decision about a health problem.” The rationale was that “health decision” might create unnecessary psychological distance for older adults with lower education levels or advanced age, while the revised phrasing aligns more closely with everyday spoken language. Expert 2 suggested modifying: “what has worked for me” to “methods that have been effective.” The rationale was that “what has worked” is a broad expression that may require more cognitive effort for older adults with lower cognitive abilities to understand its specific meaning. Experts 3 and 5 recommended modifying: “I strictly follow..” to “I follow...exactly as given.” The rationale was that “strictly follow” emphasizes attitude and often carries a tone of obedience, whereas “exactly” places greater emphasis on the degree of behavioral execution.

### 3.3 Pilot testing

Pilot testing was conducted with 30 older adults, including 17 men and 13 women, with a mean age of 73.43 ± 10.71 years. Four participants recommended replacing “healthcare personnel” with “doctors or nurses” throughout the questionnaire. They considered the former term overly technical and less familiar, particularly for participants with lower educational attainment or long-term rural residence, whereas “doctors or nurses” was perceived as clearer and more relatable.

### 3.4 Item analysis

Item performance was evaluated using the critical ratio method, item-total correlations, and changes in the Cronbach’s α coefficient of the corresponding subscale after item deletion. Participants were ranked according to the overall C-DECIDE score, and those in the upper and lower 27% were assigned to the high- and low-score groups, respectively. Independent-samples t tests showed that all items significantly discriminated between the two groups, with critical ratio values ranging from 12.644 to 26.421 (all *p* < 0.001). Item-total correlations ranged from 0.519 to 0.741 (all *p* < 0.001), supporting satisfactory item homogeneity. Deleting any item reduced the Cronbach’s α coefficient of its corresponding subscale; therefore, all 20 items were retained. Detailed results are presented in Table 3.

**Table 3:** Item analysis of the C-DECIDE.

Factor	Item	Item score	Critical ratio	Item-total correlation coefficient	*p*	Cronbach’s α if item deleted
Factor 1	1	2.828 (0.769)	23.680	0.597	<0.001	0.813
	5	3.145 (1.213)	26.421	0.584	<0.001	0.828
	6	2.757 (0.986)	22.594	0.631	<0.001	0.841
	9	2.895 (0.641)	15.221	0.588	<0.001	0.820
	19	2.701 (0.827)	18.714	0.624	<0.001	0.817
Factor 2	2	2.379 (0.914)	23.293	0.582	<0.001	0.821
	4	2.065 (0.741)	14.127	0.641	<0.001	0.803
	13	2.761 (0.837)	20.039	0.538	<0.001	0.824
	15	2.533 (0.674)	14.559	0.519	<0.001	0.790
	18	2.305 (0.787)	12.710	0.524	<0.001	0.818
Factor 3	3	3.084 (0.967)	23.293	0.732	<0.001	0.807
	7	2.760 (1.061)	12.644	0.605	<0.001	0.814
	10	2.987 (0.973)	15.317	0.580	<0.001	0.819
	12	3.142 (0.752)	18.560	0.611	<0.001	0.828
	17	2.717 (0.669)	14.415	0.593	<0.001	0.835
Factor 4	8	2.463 (0.815)	22.993	0.627	<0.001	0.864
	11	2.690 (0.913)	18.457	0.741	<0.001	0.805
	14	2.527 (0.748)	14.763	0.565	<0.001	0.847
	16	2.558 (0.837)	21.809	0.721	<0.001	0.810
	20	2.616 (0.710)	23.210	0.615	<0.001	0.871

### 3.5 Validity analysis

#### 3.5.1 Content validity

Content validity was assessed via Delphi expert consultation with five specialists. The I-CVI ranged from 0.800 to 1.000, and the S-CVI was 0.950, indicating satisfactory content validity.

#### 3.5.2 Construct validity

EFA indicated satisfactory sampling adequacy, with a KMO value of 0.937, and Bartlett’s test of sphericity was statistically significant (χ^2^ = 3875.312, *p* < 0.001). Principal axis factoring with Promax rotation identified four factors with eigenvalues greater than 1. The resulting factor structure was theoretically interpretable and consistent with the four dimensions of the original DECIDE inventory. All items had primary pattern coefficients above 0.40, while all secondary pattern coefficients were below 0.30, indicating the absence of problematic cross-loadings. The four-factor solution accounted for 65.731% of the common variance. The pattern matrix is presented in Table 4. CFA further supported the four-factor structure, demonstrating acceptable model fit: CMIN/DF = 2.513, RMR = 0.047, RMSEA = 0.028, TLI = 0.946, IFI = 0.953, CFI = 0.946, GFI = 0.948, and AGFI = 0.945. Detailed results are provided in Table 5 and Figure 1.

**Table 4:** Pattern matrix of the four-factor C-DECIDE solution.

Item	Independent decisions	Responsible decisions	Self-neglected decisions	Guided decisions
1. About my health issues, I make decisions on my own and do not need to seek opinions from others.	**0.829**	0.269	0.218	0.149
5. When I have a health problem, I decide to use my own methods instead of seeking help.	**0.786**	0.271	0.072	0.087
6. Even if medical personnel suggest a treatment plan, I may decide not to follow it and act in my own way.	**0.749**	0.212	0.184	0.082
9. I decide to take only medications I think are useful for health problems that matter to me.	**0.717**	0.231	0.203	0.217
19. I make decisions about my health based on my own experiences and what has worked for me in the past.	**0.683**	0.258	0.221	0.183
2. When I need to make a health decision, I consult healthcare personnel.	0.118	**0.796**	0.260	0.185
4. I consider carefully before making decisions about my health.	0.094	**0.649**	0.217	0.115
13. I try to collect useful information before deciding what to do about a health issue.	0.171	**0.810**	0.276	0.249
15. When I have a health problem, I take all the time I need before deciding what to do, to ensure I have understood the advice from healthcare personnel.	0.092	**0.591**	0.269	0.173
18. I consider all possible options before determining the most effective action to address a health problem.	0.084	**0.589**	0.096	0.139
3. I do not think it is important to make decisions regarding my own health.	0.115	0.085	**0.780**	0.139
7. I have decided not to take any responsibility for my health.	0.210	0.074	**0.721**	0.104
10. I do not really care about making decisions about my health.	0.202	0.187	**0.642**	0.286
12. I have decided not to follow any treatment recommended by medical personnel because I feel it is useless for my situation.	0.110	0.262	**0.597**	0.083
17. I do not care who makes decisions about my health.	0.248	0.102	**0.535**	0.113
8. When I need to make a decision about a health problem, I fully rely on healthcare personnel and do not attempt to manage it myself.	0.229	0.115	0.075	**0.875**
11. When I need to make health decisions, I seek advice from medical personnel because they know more than I do.	0.210	0.092	0.190	**0.791**
14. Which medications I need to take is decided by the doctor, I do not need to know the uses of these medications.	0.149	0.084	0.143	**0.743**
16. I prefer to let healthcare professionals, who know more than me, make decisions about my health for me.	0.210	0.145	0.127	**0.684**
20. I strictly follow the doctor’s prescription and medical personnel’s advice, without questioning or trying to understand the reasons.	0.096	0.262	0.117	**0.570**

Bold values indicate the primary pattern coefficient for each item.

**Table 5:** Model fit indices of C-DECIDE in CFA.

Model	CMIN/DF	RMR	RMSEA	TLI	IFI	CFI	GFI	AGFI
Four-factor model	2.513	0.047	0.028	0.946	0.953	0.946	0.948	0.945
Standard	<3	<0.05	<0.08	>0.9	>0.9	>0.9	>0.9	>0.9

**Figure 1:**
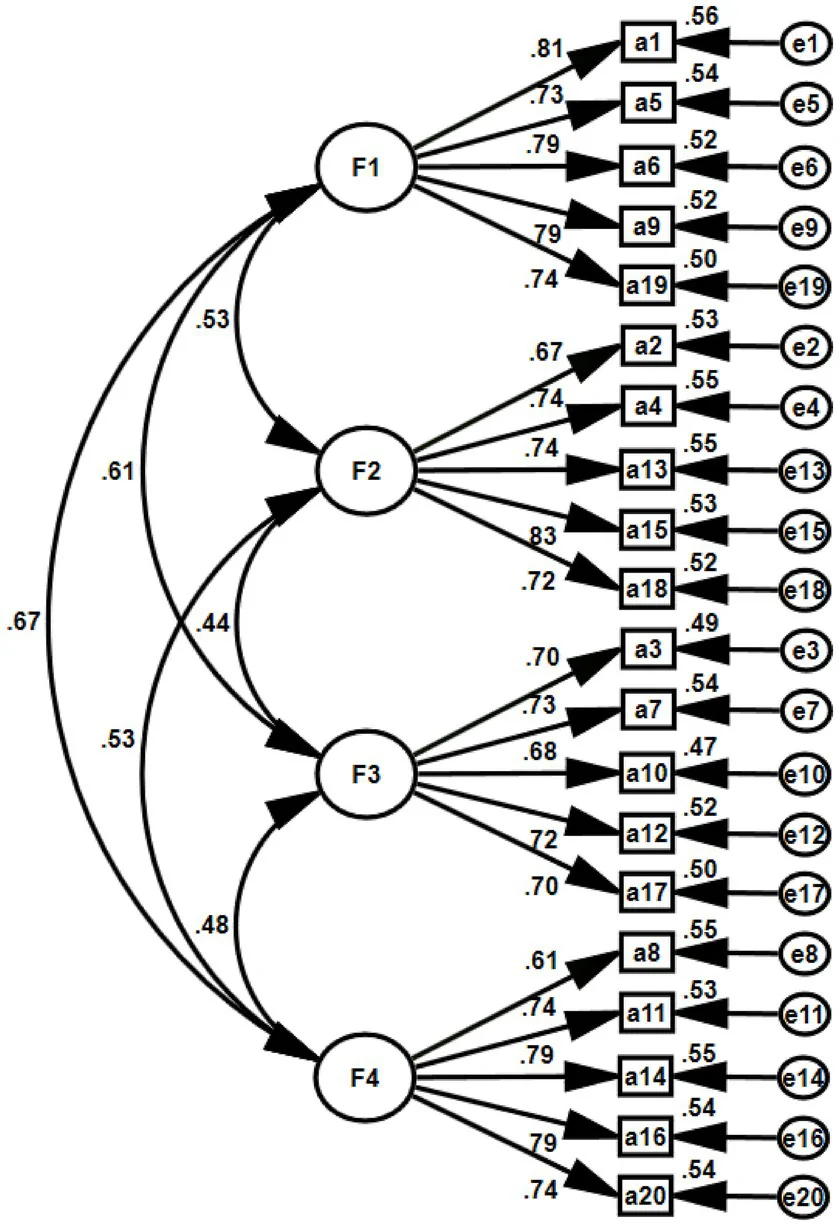
Standardized CFA model of the C-DECIDE. F1: Independent Decisions; F2: Responsible Decisions; F3: Self-Neglected Decisions; F4: Guided Decisions.

#### 3.5.3 Convergent and discriminant validity

Convergent validity was supported by AVE values of 0.502–0.588 and CR values of 0.834–0.877 across the four dimensions. Discriminant validity was also supported, as the square roots of AVE (0.709–0.767) exceeded the corresponding inter-factor correlations. Details are shown in Table 6.

**Table 6:** Convergent and discriminant validity of the C-DECIDE.

Factor	AVE	CR	Correlation between factors			
			Factor 1	Factor 2	Factor 3	Factor 4
Factor 1	0.588	0.877	0.767^**①**^			
Factor 2	0.550	0.859	0.531	0.742^**①**^		
Factor 3	0.502	0.834	0.607	0.440	0.709^**①**^	
Factor 4	0.543	0.855	0.673	0.534	0.478	0.737^**①**^

^**①**^represents the square root of the AVE value.

### 3.6 Reliability analysis

The C-DECIDE demonstrated good reliability. Internal consistency was high (Cronbach’s α = 0.896), with subscale α values ranging from 0.839 to 0.877. Split-half reliability was 0.790, with subscale coefficients ranging from 0.837 to 0.848. Test–retest reliability over a 2-week interval was 0.861, with subscale coefficients ranging from 0.853 to 0.907. Detailed results are presented in Table 7.

**Table 7:** Reliability coefficients of the C-DECIDE.

Factor	Number of items	Cronbach’s α coefficient	Split-half reliability	Retest reliability
DECIDE	20	0.896	0.790	0.861
Factor 1	5	0.848	0.842	0.853
Factor 2	5	0.853	0.848	0.856
Factor 3	5	0.839	0.837	0.879
Factor 4	5	0.877	0.841	0.907

## 4 Discussion

With the rising burden of chronic conditions and the increasing complexity of health management in later life, older adults must navigate a broad range of health-related decisions (2, 4, 5). However, existing instruments in China mainly assess disease-specific self-care behaviors or general self-management capacity and provide limited insight into how older adults characteristically approach such decisions (19–24). The DECIDE focuses on how older adults make self-care decisions rather than only on the behaviors they ultimately perform (6, 8). Its introduction and cultural adaptation may therefore provide a more targeted means of assessing self-care decision-making styles among Chinese older adults and informing individualized health education, communication, and decision-support strategies (9, 15–18).

To our knowledge, this is the first study to translate, culturally adapt, and psychometrically validate the DECIDE for use among older adults in China. The original DECIDE drew on established work on decision-making styles and self-care among older adults, with Responsible, Guided, Self-Neglected, and Independent Decisions broadly corresponding to rational, dependent, avoidant, and intuitive decision-making tendencies, respectively (6, 7). Following standardized translation, expert review, and pilot testing, the C-DECIDE retained the conceptual organization of the original instrument.

Item analysis showed that all items performed satisfactorily. They demonstrated adequate discrimination and appropriate correlations with the corresponding scale scores, and removal of any item did not improve the internal consistency of its respective dimension. These findings indicate that the translated items were sufficiently homogeneous and contributed appropriately to the assessment of self-care decision-making styles. Accordingly, all 20 items were retained.

Reliability was assessed using Cronbach’s α, split-half reliability, and test–retest reliability. The Cronbach’s α coefficient was 0.896 for the overall scale and ranged from 0.839 to 0.877 across the four dimensions, numerically higher than the corresponding internal consistency coefficients of 0.691–0.796 reported for the original DECIDE (6). This difference may partly reflect the linguistic and cultural adaptation of the instrument, which may have reduced ambiguity in item interpretation and promoted more consistent responses within each dimension (32, 33). Differences in sample composition may also have contributed. The present sample was predominantly rural, included a high proportion of older adults with low educational attainment, and most participants lived with family members. These sociodemographic and living characteristics may have influenced response variability and, consequently, the observed internal consistency estimates. Split-half reliability also supported the consistency of the C-DECIDE. The 2-week test–retest coefficients across the four dimensions ranged from 0.853 to 0.907, compared with 0.897–0.970 over a 1-week interval in the original study (6). The numerically lower test–retest coefficients observed for most dimensions in the present study may partly reflect the longer retest interval, which allows greater opportunity for changes in health status, healthcare experiences, or decision-making circumstances between assessments. Nevertheless, all four dimensions remained highly stable over the 2-week period, further supporting the reliability of the C-DECIDE in the present sample.

The validity findings provided further evidence for the psychometric performance of the C-DECIDE. Expert evaluation indicated good content validity, with the translated items considered relevant and representative of the intended content domains. EFA identified four dimensions, namely Independent Decisions, Responsible Decisions, Self-Neglected Decisions, and Guided Decisions, and CFA further supported this structure. Convergent and discriminant validity also indicated that the four dimensions reflected related but distinct aspects of self-care decision-making. Notably, this four-dimensional structure was consistent with the conceptual organization of the original DECIDE (6). However, health-related decisions among Chinese older adults often involve substantial participation from family members and healthcare professionals, which may influence how independent and guided decision-making are expressed (9, 27). Despite these cultural and contextual differences, the existing 20 items retained the original four-dimensional organization in the present study, suggesting that the four decision-making styles remained applicable in the Chinese sample. Taken together, these findings provide preliminary support for the C-DECIDE as a reliable and valid instrument for assessing self-care decision-making styles among Chinese older adults.

### 4.1 Limitations

This study has several limitations. First, rural residents accounted for 75.8% of the sample, substantially higher than the 46.0% reported among Chinese adults aged ≥60 years in a national survey (42). Similarly, 72.3% of participants had an elementary school education or below, compared with 61.0% of older adults nationally; however, this proportion was close to the 74.9% reported among rural older adults (42). Thus, the relatively low educational attainment of the sample may partly reflect its predominantly rural composition. Nevertheless, the overrepresentation of rural residents and the use of convenience sampling may limit the generalizability of the findings. Second, the present study translated and validated the existing 20-item DECIDE rather than generating new items specifically from the experiences of Chinese older adults. Therefore, although the original four-dimensional structure was supported, the present findings cannot determine whether these four dimensions encompass all self-care decision-making styles that may be relevant in the Chinese cultural context. Third, although several forms of validity were examined, predictive validity was not evaluated, and the relationships between the C-DECIDE and a broader range of theoretically and clinically relevant variables remain unclear. Fourth, although EFA and CFA were conducted in separate subsamples, both subsamples were drawn from the same overall sample, and no independent external validation sample was included. Finally, content validity was assessed by five experts whose expertise was primarily in clinical medicine and nursing, without specialists in geriatric psychology, cognitive psychology, or psychometrics. This may have limited the evaluation of the psychological and cognitive aspects of self-care decision-making styles.

### 4.2 Implications for future research

Future research should further validate the C-DECIDE in more representative and geographically, socioeconomically, and culturally diverse populations. Probability-based sampling across additional provinces, urban and rural settings, and different healthcare contexts would help clarify its generalizability. Independent external validation is also needed to examine whether the four-dimensional structure can be replicated in separately recruited samples. Qualitative and mixed-methods studies could further explore whether additional culturally specific self-care decision-making patterns exist among Chinese older adults and whether the current four-dimensional framework requires refinement. Cross-cultural measurement invariance between Chinese and original-language samples should also be examined to determine whether the four decision-making styles are interpreted and measured similarly across cultural contexts (43). In addition, measurement invariance across demographic and clinical subgroups, such as sex, age, educational attainment, residence, and chronic disease status, should be evaluated (43). Longitudinal studies are needed to determine whether C-DECIDE scores predict clinically and behaviorally relevant outcomes, including self-care behaviors, treatment adherence, healthcare utilization, and quality of life, and to further examine their relationships with theoretically relevant factors such as health literacy, frailty, social support, self-efficacy, and shared decision-making. Future cross-cultural adaptation studies may also benefit from larger and more multidisciplinary expert panels that include specialists in geriatric psychology, cognitive psychology, and psychometrics (32, 33).

## 5 Conclusion

This study translated and culturally adapted the DECIDE into Chinese and evaluated its psychometric properties in a multicenter convenience sample of older adults from four Chinese provinces. The 20-item C-DECIDE retained the original four-factor structure and demonstrated satisfactory reliability and validity. These findings provide preliminary support for its use in assessing self-care decision-making styles among older adults in the sampled regions and may inform the development of individualized decision support and nursing interventions.

## Data Availability

The datasets generated and/or analyzed during the current study are not publicly available due to data-sharing restrictions stipulated in the participant agreements, but are available from the corresponding author on reasonable request.
